# G6PD Orchestrates Genome-Wide DNA Methylation and Gene Expression in the Vascular Wall

**DOI:** 10.3390/ijms242316727

**Published:** 2023-11-24

**Authors:** Christina Signoretti, Sachin A. Gupte

**Affiliations:** Department of Pharmacology, New York Medical College, Valhalla, NY 10595, USA; csignore@student.touro.edu

**Keywords:** DNA methylation, G6PD variant, rat, human SNP, vascular biology

## Abstract

Recent advances have revealed the importance of epigenetic modifications to gene regulation and transcriptional activity. DNA methylation, a determinant of genetic imprinting and the de novo silencing of genes genome-wide, is known to be controlled by DNA methyltransferases (DNMT) and demethylases (TET) under disease conditions. However, the mechanism(s)/factor(s) influencing the expression and activity of epigenetic writers and erasers, and thus DNA methylation, in healthy vascular tissue is incompletely understood. Based on our recent studies, we hypothesized that glucose-6-phosphate dehydrogenase (G6PD) is a modifier of DNMT and TET expression and activity and an enabler of gene expression. In the aorta of CRISPR-edited rats with the Mediterranean G6PD variant, we determined DNA methylation by whole-genome bisulfite sequencing, gene expression by RNA sequencing, and large artery stiffness by echocardiography. Here, we documented higher expression of *Dnmt1, Dnmt3a*, *Tet2*, and *Tet3* in aortas from Mediterranean G6PD^S188F^ variant (a loss-of-function single nucleotide polymorphism) rats than their wild-type littermates. Concomitantly, we identified 17,618 differentially methylated loci genome-wide (5787 hypermethylated loci, including down-regulated genes encoding inflammation- and vasoconstriction-causing proteins, and 11,827 hypomethylated loci, including up-regulated genes encoding smooth muscle cell differentiation- and fatty acid metabolism-promoting proteins) in aortas from G6PD^S188F^ as compared to wild-type rats. Our results demonstrated that nitric oxide, which is generated in a G6PD-derived NADPH-dependent manner, increases TET and decreases DNMT activity. Further, we observed less large artery (aorta) stiffness in G6PD^S188F^ as compared to wild-type rats. These results establish a noncanonical function of the wild-type G6PD and G6PD^S188F^ variant in the regulation of DNA methylation and gene expression in healthy vascular tissue and reveal that the G6PD^S188F^ variant contributes to reducing large artery stiffness.

## 1. Introduction

Gene regulation is a formidable process now known to be mediated in part through DNA methylation and chromatin (histone) acetylation and methylation [1]. Moreover, the impact of these epigenetic modifications on the pathobiology of cancer and vascular disease is now apparent [2,3]. However, there are critical gaps in our knowledge regarding the epigenetic modifications (DNA methylation and histone modification) that contribute to the transformation of vascular cells from a healthy to a disease phenotype [2].

Several epigenetic modifiers, including histone deacetylases (HDACs), histone acetyltransferases (HATs), lysine demethylases (KDMs), and DNA methyltransferases (DNMTs) and demethylases (TETs), regulate gene expression. Interestingly, the activity of these epigenetic modifiers is driven by cellular metabolism [4]. For example, HDAC activity depends on NAD(P)H redox [5], while KDMs and TETs depend on α-ketoglutarate [6,7,8], and DNMTs depend on metabolites from ancillary one-carbon metabolism and polyamine pathways [9]. We and others have shown that metabolites of one-carbon metabolism and the polyamine pathway are significantly reduced within the lungs and smooth muscle cells (SMCs) of mice and humans after the inhibition of glucose-6-phosphate dehydrogenase (G6PD) activity, as well as in red blood cells from G6PD-deficient blood donors [10,11]. Nonetheless, the significance of G6PD in intermediary biochemical reactions, such as one-carbon metabolism, to gene expression is underappreciated.

We recently discovered that G6PD interacts with epigenetic modifiers (DNMTs and KDMs) in vascular tissue [10], and in the lungs of hypertensive mice, inhibiting G6PD activity decreases the expression of *Dnmt3b* while increasing the expression of *Tet2* [10,12]. Interestingly, induced pulmonary hypertension elicited a smaller increase in DNMT1 expression and activity in Mediterranean G6PD variant rats harboring a loss-of-function non-synonymous single nucleotide polymorphism (SNP; S188F; Type A-; G6PD^S188F^; severely deficient) than in their wild-type (WT) littermates [13]. At the same time, DNA hypomethylation and up-regulated expression of nitric oxide synthase 3 (NOS3) and superoxide dismutase were observed in the G6PD^S188F^ rats. In addition, epidemiological studies suggest that individuals in the Mediterranean region carrying a loss-of-function G6PD variant are less susceptible to cardiovascular disease [14,15]. DNMTs and TETs affect gene expression under both normal physiological and disease conditions. Our studies indicate that levels of two metabolites in the polyamine and one-carbon pathways, spermine and dimethylglycine (DMG), respectively, are lower in vascular tissues from G6PD^S188F^ rats than their WT littermates [16]. From those observations, we speculate that WT G6PD and the G6PD^S188F^ variant must be involved in regulating DNMT expression and activity. Up to now, however, studies have focused on how G6PD regulates the expression and activity of DNMTs and TETs under disease conditions. Its function in the normal physiological state remains unknown. Further, the extent to which G6PD controls genome-wide DNA methylation status is undetermined. In this study, therefore, we investigated the importance of G6PD to DNA methylation and gene regulation in vascular tissue from healthy WT and G6PD^S188F^ variant rats.

## 2. Results

### 2.1. The Loss-of-Function G6PD^S188F^ Variant and Differential Gene Expression

**Figure 1:**
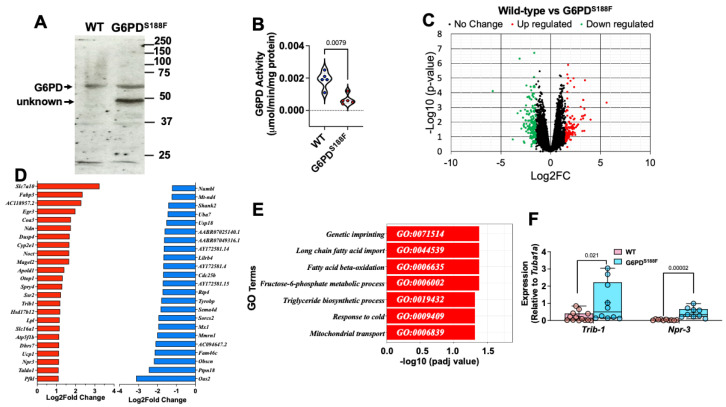
Gene expression in aortas from WT and G6PD^S188F^ rats. Nuclear extracts prepared from the aortas of G6PD^S188F^ rats show the same (**A**) G6PD expression but less (**B**) G6PD activity than aortic extracts from WT rats. (**C**) Volcano plot comparing the transcriptomic profile between WT and G6PD^S188F^ rats reveals numerous genes differentially up- or down-regulated (black circles; NS) between the two genotypes; for a few genes, the differential increase (>1.5-Log2fold; red circles) or decrease (>1.5-Log2fold; blue circles) was significant. (**D**) Top 24 increased (red bar) and 23 decreased (blue bar) genes are shown. (**E**) GO term enrichment of all significantly (−Log10 (*p*adj < 0.05)) altered genes is shown. (**F**) We confirmed the gene expression results obtained using RNAseq with qPCR and found greater expression of *Trib1* and *Npr3* in aortas from G6PD^S188F^ than WT rats. Number of rats (N) = 5 in panel (**A**,**B**); N = 6 in panels (**C**–**E**); and N = 10–14 in panel (**F**).

Within cells, G6PD is localized in several subcellular compartments, including the plasma membrane, nucleus, and cytoplasm [17,18]. Within the nucleus of hepatic cells, G6PD fuels NADPH oxidase-dependent superoxide anion production [19], which in turn regulates gene expression in endothelial cells [20]. To determine whether nuclear G6PD within the vascular cells contributes to gene regulation, we assessed its expression and activity in nuclei isolated from rat aortas collected from G6PD^S188F^ rats and their WT littermates. We detected less (65%; *p* = 0.0079) G6PD activity in nuclei from the aortas of G6PD^S188F^ than in WT aortas (Figure 1A,B), which is consistent with earlier observations in liver tissue from G6PD^S188F^ rats [16] and in humans carrying the G6PD^S188F^ variant [21].

We next performed RNAseq and found 13,801 up- or down-regulated genes in aortas from G6PD^S188F^ (*n* = 6) as compared to WT (*n* = 6) rats (Figure 1C). However, 1509 out of 13,801 genes were significantly increased (red circle; >1.5Log2Fold Change; *p* < 0.05) or decreased (blue circle; >1.5Log2Fold Change; *p* < 0.05). Top significantly up (Figure 1D; red bars; *p*adj < 0.05)- and down (Figure 1D; blue bars; *p*adj < 0.05)-regulated genes are shown. The GO term enrichment analysis of all significantly (−Log10 (*p*adj < 0.05)) up- and down-regulated genes indicated that the genes were related to imprinting, long-chain fatty acid import, fatty acid β-oxidation, fructose-6-phosphate metabolic and triglyceride biosynthetic processes, response to cold, and mitochondrial import pathways (Figure 1E). Further, GO term enrichment indicated that the top increased genes were associated with the deadenylation-dependent decapping of nuclear-transcribed mRNA, the negative regulation of transcription, and SMC proliferation pathways. Conversely, GO term enrichment indicated the top decreased genes were related to RNA catabolism, DNA damage, and the positive regulation of cell cycle G2/M phase transition pathways. To validate the RNAseq results, we selected *Trib-1* and *Npr-3* because they encode proteins that control critical signal transduction in SMC proliferation [22,23]. We performed qPCR and confirmed that *Trib-1* and *Npr-3* were up-regulated in aortas from G6PD^S188F^ as compared to WT rats (Figure 1F).

### 2.2. The Loss-of-Function G6PD^S188F^ Variant Led to Augmented Expression of Genes Encoding DNA Methylases and Demethylases

**Figure 2:**
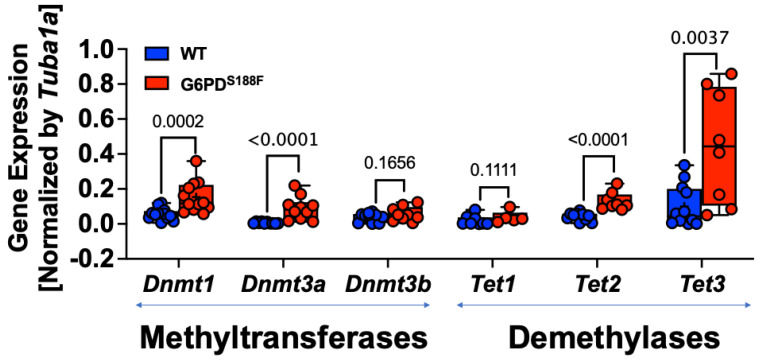
Expression and activity of DNMTs and TETs in aortas from WT and G6PD^S188F^ rats. Determination of *Dnmt* (1, 3a, and 3b) and *Tet* (1, 2, and 3) gene expression by qPCR revealed higher expression of *Dnmt1, Dnmt3a, Tet2, and Tet3* in aortas from G6PD^S188F^ than WT rats.

Because the RNAseq results revealed that the expression of imprinted genes (*Ndn* and *Magel2*) was higher in aortas from G6PD^S188F^ than WT rats (Figure 1) and that the expression as well as activity of DNA methylases, which are implicated in imprinting [24], are regulated in a G6PD-dependent manner in the lungs of rats and mice [10,12,13], we assessed the expression by qPCR and the total activity of DNA methylases (DNMT-1, -3A, and -3B) and demethylases (ten-eleven translocase (TET)-1, -2, and -3). We detected 2-fold higher expression of *Dnmt1* (*p* = 0.0002) and *Dnmt3a* (*p* < 0.0001) in aortas from G6PD^S188F^ than WT rats (Figure 2). Our results also revealed higher expression of *Tet2* (2-fold; *p* < 0.0001) and *Tet3* (4-fold; *p* = 0.0037) in G6PD^S188F^ than WT aortas (Figure 2).

### 2.3. DNMT and TET Activity Correlates with Nitric Oxide and Nitric Oxide Synthase Inhibitor Augmented DNMT and Attenuated TET Activity in Aorta

**Figure 3:**
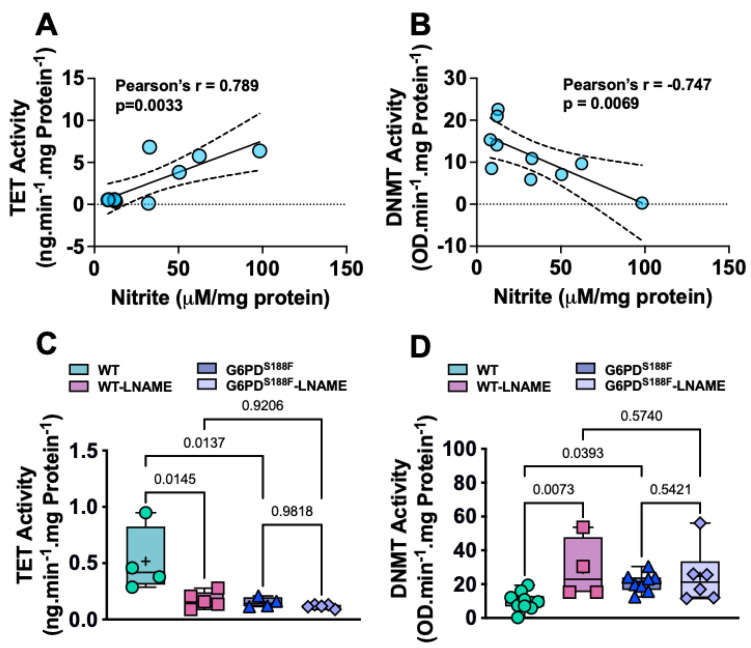
DNMT and TET activity is regulated by nitric oxide in aortas from WT and G6PD^S188F^ rats. (**A**,**B**) A positive and negative correlation between TET and DNMT activity, respectively, with endogenous nitric oxide metabolite nitrite levels indicates that nitric oxide, at least partly, controls their activity. (**C**,**D**) L-N^G^-nitroarginine methyl ester (L-NAME), which inhibits nitric oxide synthase activity and reduces nitric oxide production, significantly decreased TET and increased DNMT activity in the aorta of wild-type (WT) rats but not G6PD^S188F^ variant rats. The activity of TET and DNMT was significantly reduced and elevated, respectively, in the aorta of control G6PD^S188F^ rats as compared with the age-matched WT littermates.

It is known that the metabolites of ancillary one-carbon metabolism and the polyamine pathway are critical to DNA methylation and gene regulation [9]. Because DNMT and TET activities are regulated by metabolites of the one-carbon pathway and the Krebs cycle, we speculated that altered activity in the one-carbon pathway and Krebs cycle must be contributing to the respective gain and loss of DNMT and TET activity observed in G6PD^S188F^ as compared to WT aortas. We previously showed that α-ketoglutarate—a metabolite of the Krebs cycle and co-factor for TET—is lower in aortas and carotid arteries from G6PD^S188F^ rats [16]. We found higher (1.4-fold) levels of methionine, a one-carbon metabolite, and higher methionine-to-dimethylglycine (DMG) ratios in aortas from G6PD^S188F^ than WT rats. Some recent studies suggest that nitric oxide (NO), a critical regulator of vascular function, controls the activity of DNMT and TET enzymes [25]. Since G6PD-derived NADPH fuels nitric oxide synthase to produce NO [26] and the G6PD^S188F^ variant decreases the generation of NO in the aorta of rats [16], to determine a potential link between the loss-of-function G6PD^S188F^ variant and activity of epigenetic modifiers, we performed correlation studies between NO and the steady-state activity of DNMT and TET enzymes. We found that TET and DNMT activity positively and negatively, respectively, correlated with nitrite levels (Figure 3A,B). Further, to confirm that endogenous NO controls DNMT and TET activity in in vivo, we treated rats with L-NAME (1 mg/mL), a NO synthase inhibitor, and then determined DNMT and TET activity in the aorta. Interestingly, we found that L-NAME (1 mg/mL) given to rats in drinking water for 5 days decreased TET activity (Figure 3C) and increased DNMT activity (Figure 3D) in the aorta of WT rats. While TET and DNMT activity decreased and increased, respectively, in the aorta of G6PD^S188F^ rats, L-NAME had no significant effect on their activity (Figure 3C,D).

### 2.4. The Loss-of-Function G6PD^S188F^ Variant Led to Decreases in Methylated Cytosine

**Figure 4:**
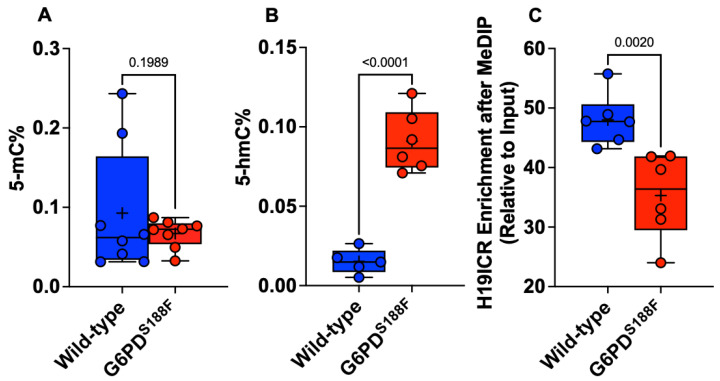
5-hmC content increased and methylation of the H19-ICR sequence decreased in aortas from G6PD^S188F^ rats. Determination of global methylation showed that (**A**) 5-mC content did not change and (**B**) 5-hmC content increased in the aortas from G6PD^S188F^ as compared to WT rats. (**C**) Methylated DNA immunoprecipitation (MeDIP) assays revealed decreased methylation of the H19-ICR sequence in aortas from G6PD^S188F^ as compared to WT rats.

The biological significance of DNA methylation as a major epigenetic modification affecting gene expression and cell phenotype is now well recognized. We therefore assayed global methylation levels by measuring levels of 5-methylcytosine (5-mC), a methylation mark, and 5-hydroxymethylcytosine (5-hmC), a demethylation mark, in aortic tissue from WT and G6PD^S188F^ rats. Because we found higher DNMT and lower TET activities in G6PD^S188F^ than WT aortas, we anticipated seeing higher 5-mC and lower 5-hmC levels in G6PD^S188F^ aortas. On the contrary, however, we found no change in 5-mC and significantly higher 5-hmC content in G6PD^S188F^ than WT aortas (Figure 4A,B). It is noteworthy that the average 5-mC (0.093 ± 0.028%)-to-5-hmC (0.015 ± 0.03%) ratio was 6.0-fold higher in the aorta of WT rats. Moreover, MeDIP assays revealed less methylation of the imprinting control region (ICR) sequence of the non-coding H19 gene in G6PD^S188F^ than WT aortas (Figure 4C). These findings indicate that there may be more hypomethylated than hypermethylated loci/regions genome-wide in G6PD^S188F^ than WT aortas. Therefore, to assess the differential methylation of loci/regions genome-wide, we performed unbiased whole-genome bisulfite sequencing (WGBS) in aortas from G6PD^S188F^ and WT rats.

### 2.5. The Loss-of-Function G6PD^S188F^ Variant Evoked Differential Methylation of DNA

**Figure 5:**
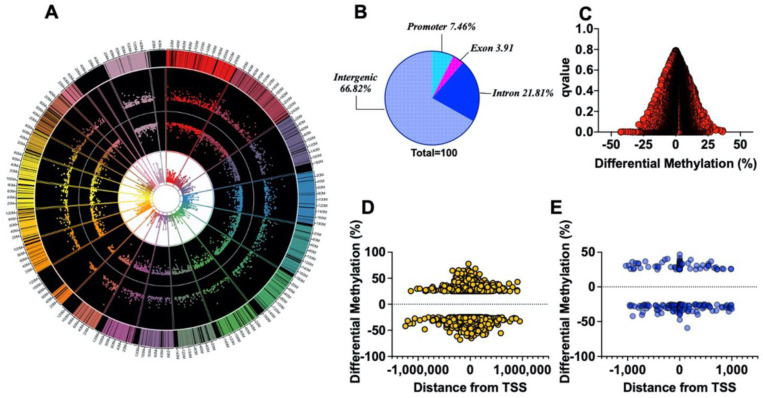
Methylome landscape in aortas from WT and G6PD^S188F^ rats. (**A**) WGBS in WT (*n* = 3) and G6PD^S188F^ (*n* = 3) rats revealed differential methylation of >17,000 loci genome-wide; the circos plot demonstrates differential methylation of loci on each chromosome (outer track), % hyper- and hypomethylation (second and third tracks from outside, respectively), and the *p*adj-value (third and innermost track from outside; min: 2.63 × 10^−120^ and max: 0.009858283). (**B**) Pie chart demonstrating differential methylation of the indicated regions within the genome. Scatter plots demonstrate differential methylation of (**C**) promoter regions (Red Circles) and (**D**) the distance (±1,000,000 kb; Yellow Circles) from the transcription start site (TSS) within the genomes of WT and G6PD^S188F^ rats. (**E**) A total of 201 loci were hypo (−25 to −59% differential methylation)- or hyper (25 to 46% differential methylation)-methylated within a 1 kbp (Blue Circles) span from TSS.

WGBS revealed 17,618 loci (of which 32% were hypermethylated and 78% were hypomethylated) and 4222 regions (of which 42% were hypermethylated and 58% were hypomethylated) containing CpG islands that were differentially methylated between G6PD^S188F^ (*n* = 3) and WT (*n* = 3) aortas. The top 100 differentially methylated annotated loci and regions are shown in Supplementary Tables S1 and S2. The circos plot shows the start and e nd segments of the differentially methylated regions on each chromosome (outer track), percent hyper- and hypomethylation (second and third track from outside, respectively), and the *p*-value (third and innermost track from outside), in G6PD^S188F^ versus WT aortas (Figure 5A). Sequencing data then revealed that genome-wide differential methylation occurred within intergenic regions, introns, exons, and promoter regions (Figure 5B). CpG islands reside within gene promoters [27]. Interestingly, among 34,525 genes examined genome-wide, we detected ≥25% methylation in very few promoter regions (hyper (0.037%)- and hypo (0.089%)-methylation; q-value < 0.05) (Figure 5C). Although hyper- and hypomethylation spanned 1,000,000 bp up- and downstream from the transcription start site (TSS or distance from feature; Figure 5D), biologically relevant methylation changes (−59% to 46%) occurred within 1000 bp up- and downstream from the TSS of 201 loci in the genome of which 65.2% were hypomethylated and 34.8 were hypermethylated (Figure 5E). GO term enrichment analysis indicated that hypermethylated (>25% and q-value < 0.05; Supplementary Figure S1A red bars) genes were related to transporter activity, molecular function regulators and transducers, protein binding activity, and catalytic activity pathways (Supplementary Figure S1B), while hypomethylated (>25% and q-value < 0.05; Supplementary Figure S1A blue bars) genes encoded proteins encompassing cellular, multicellular organismal, metabolic, localization, signaling, and developmental processes (Supplementary Figure S1C).

### 2.6. The Loss-of-Function G6PD^S188F^ Variant Led to Hypomethylated and Augmented Genes Encoding Proteins Involved in Metabolic Processes, Cold Thermogenesis, and Reactive Oxygen Biosynthetic Processes

**Figure 6:**
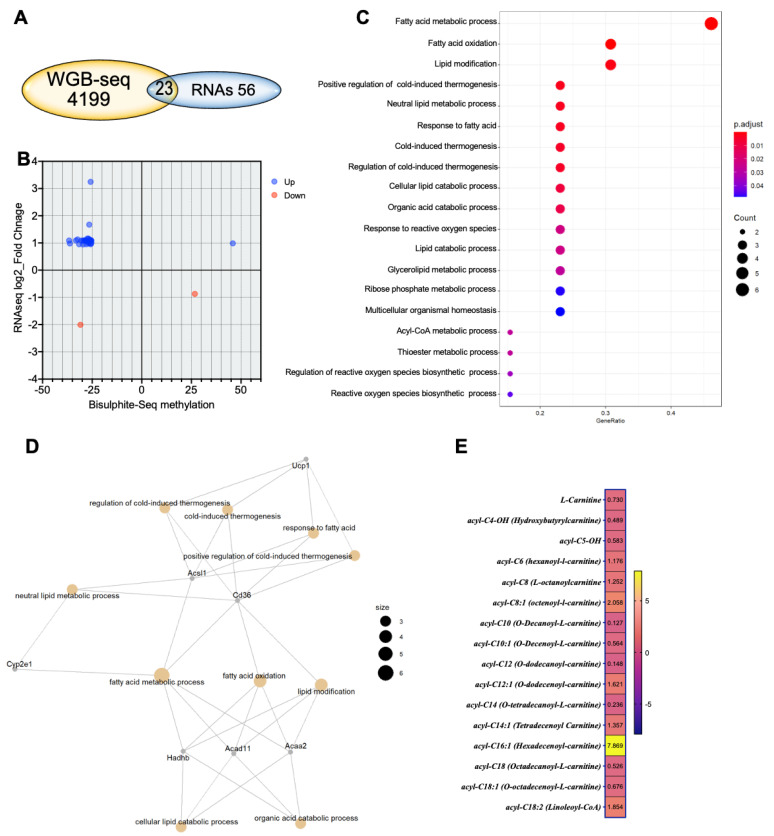
Integration of WGBS and RNAseq results reveals that genes related to metabolism and reactive oxygen species generation are affected by the G6PD^S188F^ variant. (**A**) Venn diagram showing the genes overlapping between the WGBS (differential tiles) and RNAseq data. (**B**) Thirty-two genes were differentially methylated and expressed (*p*adj < 0.05); of those, 29 were hypomethylated, and their expression was increased. (**C**) GO term enrichment analysis indicating that the hypomethylated and increased gene sets were related to fatty metabolism, lipid modification, lipid and organic acid catabolic processes, cold thermogenesis, ribose phosphate metabolic processes, multicellular organismal homeostasis, and reactive oxygen species biosynthetic processes. (**D**) Connections between the GO terms and the gene set were detected through string analysis. (**E**) Metabolomic analysis revealed that fatty acid metabolites and acyl-coA products regulated by *Acsl1*- and *Acad11*-encoded proteins were significantly altered in aortas from G6PD^S188F^ rats.

Genes revealed to be differentially methylated between G6PD^S188F^ and WT aortas in the WGBS analysis were cross-referenced with their gene expression profiling. The Venn diagram in Figure 6A shows the genes overlapping between the WGBS (differential tiles) and RNAseq data (differentially expressed genes, *p*adj < 0.05 and at least 2-fold change). To expand the data included in the analysis, we next included all genes that were differentially expressed (*p*adj < 0.05) and were also differentially methylated (>25% and q-value < 0.05) in the WGBS data. That resulted in 32 genes for downstream analysis, among which the expression of 29 hypomethylated genes was increased (Figure 6B). GO term enrichment analysis of the genes that were up-regulated and had lower methylation revealed the enriched terms to be related to fatty acid metabolism, lipid modification, lipid and organic acid catabolic processes, cold thermogenesis, ribose phosphate metabolic processes, multicellular organismal homeostasis, and reactive oxygen species biosynthetic processes (Figure 6C). The connection between the GO terms and the gene set was obtained through string analysis (Figure 6D). Correspondingly, fatty acid metabolites and acyl-coA products regulated by proteins encoded by *Acsl1* and *Acad11* were significantly altered in aortas from G6PD^S188F^ as compared to WT rats (Figure 6E).

### 2.7. The Loss-of-Function G6PD^S188F^ Variant Led to the Hypomethylation of Genes Encoding Negative Regulators of SMC Proliferation and Steroid Biosynthesis and Reduced Large Artery Stiffness

**Figure 7:**
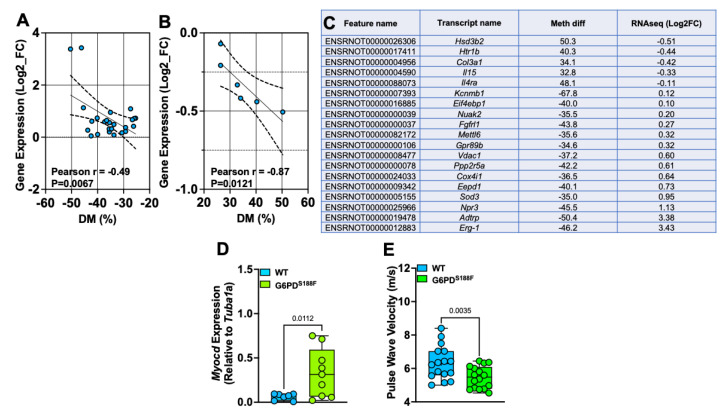
SMC phenotype- and function-related genes are differentially methylated and expressed between aortas from WT and G6PD^S188F^ rats. (**A**,**B**) In the aorta, gene expression negatively correlated with the % hypomethylation and hypermethylation of the loci. (**C**) Percent methylation and Log2fold expression of genes related to SMC phenotype and function are shown. (**D**) qPCR results showing increased expression of hypomethylated *Myocd* in aortas of G6PD^S188F^ as compared to WT rats. (**E**) Echocardiography results showing less pulse wave velocity in G6PD^S188F^ as compared to WT rats.

To determine whether the G6PD^S188F^ variant leads to the modification of the methylation profiles of genes involved in SMC function and/or growth, we examined the correlation between the WGBS and RNAseq data (Figure 7A,B). Our results show a strong negative correlation between differential methylation (%) and gene expression. Genes related to SMC contraction (5-hydroxytryptamine receptor 1B: *Htr1b*) and SMC remodeling/inflammation (collagen 3 alpha1: *Col3a1*; and interleukin-15: *Il15*) were hypermethylated in G6PD^S188F^ as compared to WT aortas (Figure 7C). RNAseq analysis indicated that the expression of hypermethylated *Col3a1* was slightly decreased. Consistently, qPCR analysis showed that *Htr1b* (WT: 0.005 ± 0.001 and G6PD^S188F^: 0.004 ± 0.002) and *Col3a1* (WT: 32.7 ± 4.7 and G6PD^S188F^: 16.7 ± 4.4; *p* = 0.034) expression was decreased. On the other hand, genes related to the negative regulation of SMC proliferation (natriuretic peptide receptor 3: *Npr-3*) and anticoagulation (androgen-dependent TFPI-regulating protein: *Adtrp*) were hypomethylated and up-regulated in G6PD^S188F^ aortas (Figure 7C). qPCR performed to confirm the RNAseq results revealed higher expression of hypomethylated *Npr-3* in aortas from G6PD^S188F^ as compared with WT rats (Figure 1F). Further, the expression of another hypomethylated gene, *Myocd* (−37.5%; q-value: 0.042), which encodes a transcription coactivator of serum response factor that prevents the dedifferentiation/proliferation of SMC, was increased (*p* = 0.012) in G6PD^S188F^ as compared to WT aortas (Figure 7D). Next, to determine the consequences of decreased inflammatory genes and increased SMC differentiated phenotype and reactive oxygen species scavenger genes on vascular function, we measured large artery stiffness. Interestingly, G6PD^S188F^ variants as compared to WT rats had significantly less PWV (Figure 7E).

## 3. Discussion

This study exposed a heretofore unknown role of the G6PD^S188F^ variant connected to DNA methylation and gene regulation in vascular tissue from healthy rats. Specifically, the G6PD^S188F^ variant led to hypomethylation and the upregulation of genes associated with mitochondrial and fatty acid metabolism, cancer suppression, multicellular organismal and development processes, and SMC differentiation. Conversely, the variant led to hypermethylation and the downregulation of genes linked to inflammation, cell proliferation, and SMC dedifferentiation. Further, our results suggest the G6PD^S188F^ variant mediated genome-wide differential methylation that activated or repressed gene transcription in all cell types comprising vascular tissue. Notably, it activated a constellation of genes associated with the differentiation of the SMC phenotype. DNA methylation, undoubtedly an important epigenetic mark of transcriptional activity, occurs in all stages of the life cycle. It is associated with the genetic imprinting (inherited DNA methylation) and gene silencing (de novo DNA methylation) that underlies the diverse gene expression profiles seen in the varied cells and tissues that make up multicellular organisms [28]. The methylation of CpG islands residing within promoters results in stable silencing of gene expression during development and differentiation [29,30,31,32]. Although DNA methylation/demethylation is dynamic and continuously ongoing in cells, the regulatory factors governing temporal control of methylation/demethylation and, in turn, gene expression are not well understood. In that regard, the inhibition of G6PD activity has been shown to regulate the transcription of circadian clock-associated genes whose expression changes in a time-dependent manner [33]. We found that imprinted *Magel2*, which encodes a protein required for proper regulation of the circadian clock [34,35], was up-regulated in healthy vascular tissue from G6PD^S188F^ rats. Further, it has been proposed that a dysfunctional circadian clock contributes to the development of vascular disease [36]. Consequently, our findings imply that G6PD is a critical contributor to the regulation of time-based DNA methylation and gene regulation and that an imbalance in the expression or activity of G6PD-dependent DNA methylation writers and erasers may contribute to the transformation of vascular cells from a healthy to a disease phenotype [2]. In addition, these findings clarify, at least partially, why inhibiting G6PD activity or silencing *G6pd* reduces SMC dedifferentiation and the severity of some types of vascular disease, which we observed previously [10,12,17,37,38,39].

DNMT-catalyzed methylation of cytosine within CpG islands in various regions and loci within the genome regulates gene expression by recruiting proteins implicated in gene repression or by obstructing the binding of transcription factor(s) to the DNA [28]. We recently reported that DNMT1 expression and activity are increased in the lungs of WT rats with induced pulmonary hypertension, but that effect was suppressed in G6PD^S188F^ variant rats [13]. While *Dnmt1* was expressed 4- and 80-fold more than *Dnmt3a* and *Dnmt3b*, respectively, in vascular tissue from WT rats, *Dnmt1* and *Dnmt3a* expression was higher in G6PD^S188F^ than WT rats. Although there may be several reasons (e.g., increases in transcriptional activity or post-translational stability) for a selective *Dnmt1*/DNMT1 and *Dnmt3a*/DNMT3A increase in vascular cells of G6PD^S188F^ rats, our observations that 5-mC levels did not change and that the percentage of hypermethylated genes was lower than hypomethylated genes in the genome of G6PD^S188F^ rats as compared to WT rats suggest that the increased DNMT3A and DNMT1 potentially maintained the hypermethylation of selective regions or loci within the genome of G6PD^S188F^ rats and down-regulated some but not all genes associated with inflammation and cell proliferation in vascular cells of G6PD^S188F^ rats. We previously showed that G6PD and cytosine-5-methyltransferase form a complex within arterial tissue [10]. We therefore propose that the S188F mutation, which is thermodynamically unstable and exhibits decreased thermal stability [21] likely alters the G6PD-DNMT3A/DNMT1 interaction and facilitates the recruitment of DNMT3A/DNMT1 to target regions within the genome of G6PD^S188F^ rats. Studies have identified numerous DNMT-containing complexes involved in regulating DNA methylation and recruiting DNMT to the DNA [24]. This highly diverse group of complexes plays a key role in imprinting and gene silencing; for example, DNMT1-containing complexes are required for maintaining global DNA methylation following DNA replication, while DNMT3A-containing complexes evoke de novo DNA methylation [24]. However, the mechanism(s) by which the DNA methyltransferases and demethylases (epigenetic writers and erasers, respectively) responsible for the differential methylation of genes are recruited to specific genomic sites remains a mystery. In addition, underlying the augmented *Dnmt1*/*Dnmt3a* transcription seen in G6PD^S188F^ rats was increased DNMT activity. We propose that increases in DNMT1/DNMT3A expression and levels of methionine, a precursor of the substrate that fuels DNMT activity, together contributed to the increase in DNMT activity seen in aortas from G6PD^S188F^ rats. Collectively, then, our findings indicate, for the first time, that in healthy vascular tissue, the loss-of-function G6PD^S188F^ variant enhances *Dnmt1*/*Dnmt3a* expression and DNMT activity leading to the downregulation of some but not all genes and potentially contributes to normal vascular cell physiology and function.

Active DNA demethylation of 5-mC catalyzed by TET enzymes is continuously ongoing within cells [40,41]. Our data revealed higher expression of *Tet2* and *Tet3* alongside increased 5-hmC and more hypo- than hypermethylated loci and promoter regions in aortas from G6PD^S188F^ rats. These hypomethylated loci encompassed genes such as *Myocd* and *Kcnmb1*, which encode pro-contractile proteins [38]; *Sod3*, which encodes an extracellular SOD that scavenges superoxide radical and protects vascular function [42]; *Erg1(Sqle)*, which encodes squalene monooxygenase, which regulates cholesterol biosynthesis and has been associated with favorable outcome in terms of fibrous cap thickness and cholesterol efflux capacity [43]; *Npr3*, which encodes a receptor that regulates blood pressure and inhibits SMC growth [23,44]; and *Adtrp*, which encodes a hydrolase activity-enabling protein that prevents coronary artery disease and blood coagulation or clotting [45,46]. We concurrently detected higher expression of those hypomethylated genes in G6PD^S188F^ than WT aortas. These unprecedented observations suggest that the elevated expression of TET2 and TET3 leads to their recruitment to selective regions of the genome in G6PD^S188F^ rats. In that regard, results from an earlier study suggest that TET2 binds to CArG-rich regions of active SMC contractile promoters (MYOCD, SRF, and MYH11), and the overexpression of TET2 augments the 5-hmC epigenetic landscape and contractile gene expression and attenuates intimal hyperplasia induced by wire injury to mouse femoral artery [47]. Conversely, TET2 knockdown exacerbates vascular injury, and the loss of TET2 and 5-hmC correlates with human atherosclerotic disease [47] and coronary allograft vasculopathy [48]. Those studies suggest that TET2 coordinates SMC phenotypic modulation through opposing effects on chromatin accessibility at the promoters of pro-contractile- versus dedifferentiation-associated genes [47]. Further, DNA methylation has been shown to prevent the differentiation of non-vascular cells [49]. Here, we demonstrate that the G6PD^S188F^ loss-of-function mutation led to augmented TET2 and TET3 expression and, in turn, hypomethylation, which activated the transcription of the SMC differentiation gene program, as well as hypermethylation, which repressed genes associated with cell inflammation and proliferation in vascular tissue. This presumably prevents the transitioning of SMCs from a healthy to a disease state and perhaps reduces susceptibility to vascular diseases such as hypertension, large artery stiffness, and intimal hyperplasia in G6PD^S188F^ rats [16,50].

Next, in pursuit of determining the mechanisms that up- and down-regulate DNMT and TET activity, respectively, in the aortas of G6PD^S188F^ variant rats, we uncovered the role of NO, whose production depends on G6PD-derived NADPH [26] and is decreased in the aorta of G6PD^S188F^ rats [16], in regulating DNMT and TET activity in vascular tissue. Our results suggested that NO increases TET activity and decreases DNMT activity because nitrite levels positively and negatively correlate with the TET and DNMT activity, respectively. This notion is confirmed by our findings that the TET activity decreased and DNMT activity increased in the aorta of wild-type rats treated with L-NAME, a NO synthase inhibitor, for five days. L-NAME neither decreased TET activity nor increased DNMT activity that was already attenuated and augmented, respectively, in control G6PD^S188F^ as compared with wild-type rats. This suggests that impaired NO production in the aorta tissue of G6PD^S188F^ rats potentially decreased steady-state TET activity and increased steady-state DNMT activity. Although the application of NO to nuclear extract protein increases DNMT activity [51], it has been observed that NO induces a global decrease in 5-mC in a murine squamous cell carcinoma model [52]. These findings corroborate our results. Therefore, altogether, these results suggest that NO plays a critical role in regulating the activity of epigenetic modifiers, and we propose that reduced NO likely increases DNMT and decreases TET activities simultaneously in the aorta of G6PD^S188F^ rats.

Although we detected higher DNMT and lower TET steady-state activity in aortas from G6PD^S188F^ than WT rats, the analysis of global DNA methylation, MeDIP, and WGBS revealed more hypo- than hypermethylation genome-wide in G6PD^S188F^ rats. This observation is perplexing but not unprecedented. Lower TET activity indicates a lower V_max_ and a slower rate of the conversion of 5-mC (substrate) to 5-hmC (product), while increased 5-hmC levels indicate augmented TET2 and TET3 expression, which together convert 5-mC to 5-hmC over a period, accumulating more in the genomic loci/regions of G6PD^S188F^ than WT rats. Additionally, previous studies have shown that active DNMTs are capable of demethylating DNA depending on the local chromatin microenvironment [53]. Interestingly, in vitro studies suggest that the application of increasing H_2_O_2_ concentrations to recombinant mammalian DNMTs switches the enzyme activity from methylase to demethylase, with H_2_O_2_ inhibiting the DNA methylating activity of DNMTs by oxidizing the cysteine residues within the catalytic site [53,54]. However, the physiological relevance of this switch remains unclear. We propose that the altered redox status in vascular cells of G6PD^S188F^ rats likely led to the oxidation of the DNMTs and demethylation of the DNA. The loss-of-function G6PD^S188F^ variant increases oxidation conditions because of a decrease in the NADPH redox state and decreased clearance of H_2_O_2_ [16]. It is also well-known that G6PD-derived NADPH controls intracellular levels of H_2_O_2_ and that G6PD knockdown or inhibition increases H_2_O_2_. We further propose that the increased activities of DNMTs together with increased expression of TET2 and TET3 evoked DNA demethylation, leading to more hypo- than hypermethylation of selective loci in the genome where there are increased oxidative conditions, such as within the arteries of G6PD^S188F^ variant rats. By contrast, loci/regions in the genome not targeted by DNMTs and TET2/TET3 likely remained methylated/hypermethylated.

As expected, gene expression negatively correlated with methylation levels, and RNAseq and qPCR analyses revealed that the expression of hypomethylated *Sod3*/*Npr3*/*Myocd* increased. By contrast, RNAseq and qPCR results showed small decreases in the expression of hypermethylated *Htr1b* and *Col3a1* in aortas from G6PD^S188F^ as compared to WT rats. These findings suggest that the G6PD-dependent level of methylation occurring in CpG regions regulated the transcriptional activity and expression of genes related to SMC function.

We previously proposed that G6PD contributes to the regulation of vascular function and SMC phenotype [37,38,55]. In those studies, we showed that increased G6PD expression and activity reduces and G6PD inhibition or knockdown augments the expression of *Myocd* and the SMC-restricted gene program [37]. Moreover, in endothelial cells, G6PD regulates gene expression and is required for establishing vascular mural cell coverage of the dorsal aorta and blood vessel maturation during early development in both zebrafish and mice [56]. These results underscore the significance of G6PD to the SMC phenotype, physiology, and function during development and during the post-development/adult stages of life.

The biochemical reactions and enzymes, including G6PD, that constitute the pentose phosphate pathway are, evolutionarily speaking, very old and have likely functioned within organisms since the earliest stages of evolution [57]. G6PD-derived NADPH, the fulcrum of redox balance, is the *sine qua non* that protects cells from oxidative stress damage and is a cofactor for numerous cellular reactions [57]. The roles of NADPH in regulating glutathione and thioredoxin redox, NADPH oxidases, which are major sources of reactive oxygen species, thiol redox-based signaling molecules such as protein kinase G and nitric oxide synthase, and fatty acid and cholesterol synthesis are well studied [38,57,58,59,60,61]. However, although the importance of subcellular G6PD and NAD(P)H redox in cardiovascular function and disease is becoming more apparent, it remains poorly studied and poorly understood [62,63].

**Figure 8:**
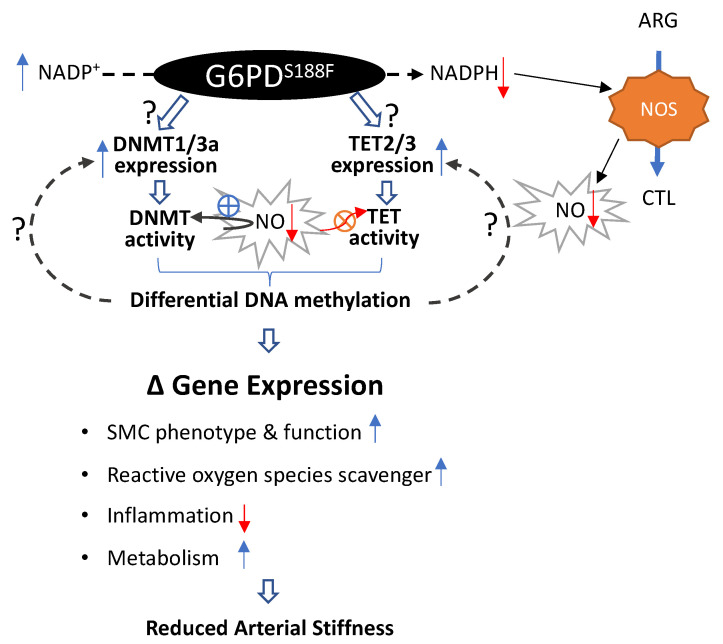
Schematic summary of the findings. A potential mechanism through which the loss-of-function G6PD^S188F^ variant regulates DNA methylation and gene expression is shown. Although G6PD-dependent mechanisms/pathways responsible for upregulating DNMT and TET expression are still unknown, NADP^+^/NADPH redox changes and decreases in G6PD/NADPH-dependent nitric oxide synthase (NOS)-derived nitric oxide (NO) augmented DNMT and attenuated TET activities in the aorta. This led to differential DNA methylation and the expression of genes encoding proteins involved in smooth muscle cell (SMC) phenotype and function, reactive oxygen species scavengers, inflammation, and metabolism. ARG: l-arginine and CTL-l-citrulline. Question marks indicate potential pathways, blue arrows indicate increase, red arrows indicate decrease, red circle with X indicate inhibition, and blue circle with + indicate activation.

The results of this study have established a noncanonical function of the wild-type G6PD and G6PD^S188F^ variant in regulating the expression and activity of nuclear DNMT and TET and in evoking the selective hyper- and hypomethylation of loci/promoter regions genome-wide that, respectively, repressed and activated genes detrimental and beneficial to vascular cell phenotype and function (Figure 8). Consistently, we observed less large artery stiffness, which is an emerging independent risk factor for cardiovascular diseases and an attractive therapeutic target [64], in G6PD^S188F^ rats as compared with WT rats. Further, although we cannot rule out a direct action of G6PD on the expression level of *Dnmt1*/*Dnmt3a*/*Tet2*/*Tet3*, we demonstrated that G6PD, at least partially through NO, regulated DNMT and TET activity, which led to differential DNA methylation and the expression of epigenetic modifiers in vascular tissue. These findings may have a significant bearing on vascular health, as the loss of G6PD function may prevent the transformation of vascular cells from a healthy to a disease phenotype. As there are several G6PD SNPs in different ethnic groups worldwide, future studies in other ethnic G6PD variant models will be needed to confirm our findings in Mediterranean G6PD^S188F^ rats and determine whether the G6PD-dependent regulation of the methylome is an SNP-specific phenomenon.

## 4. Materials and Methods

### 4.1. Animal Models and Experimental Protocols

To determine the expression and activity of DNA methylation writers and erasers in the aorta, we used loss-of-function Mediterranean G6PD^S188F^ variant rats (male: 450–950 g) and their age-matched WT littermates [16]. Some rats were treated with L-N^G^-nitroarginine methyl ester (L-NAME; 1 mg/mL in drinking water) for 5 days. Aortas were harvested and used to perform whole genome bisulfite sequencing, RNAseq, qPCR, and other biochemical analyses. Data analysis was performed in a blinded fashion.

### 4.2. Echocardiography

Echocardiography was performed in 2% isoflurane-anesthetized rats using a Vevo 770 imaging system (VisualSonics, Toronto, ON, Canada). Briefly, Pulse wave velocity (PWV) was determined from transit time between Doppler flow signals in the carotid and iliac arteries. Doppler signals and electrocardiogram (ECG) were recorded simultaneously, and the data were stored for subsequent off-line analysis. as described previously [65].

### 4.3. Preparation of Nuclear Extracts and Measurement of Global DNA Methylation and Total DNMT and TET Activity

Tissue samples were pulverized in liquid nitrogen, and nuclear extracts were prepared per the manufacturer’s instructions using a kit (Cat # OP-0002-1) from EpiGentek (Farmingdale, NY, USA). The collected nuclear extracts (20 μg protein) were then used to measure global DNA methylation and DNMT and TET activities with kits from EpiGentek. Global DNA methylation marks, 5-methylcystosine and 5-hydroxymethylcytosine, were measured by the MethylFlashTM Global DNA Methylation (5-mC) ELISA Easy Kit (Cat # P-1030) and the MethylFlashTM Global DNA Hydroxymethylation (5-hmC) ELISA Easy Kit (Cat # P-1032) from EpiGentek. Both these kits are widely used to determine 5-mC [66] and 5-hmC [67], and 5-mC and 5-hmC levels determined by these methods in samples of different organs from various species closely correlate with those obtained by HPLC-MS and MS-LC as per the manufacturer’s datasheet. DNMT and TET activities were determined by the EpiQuik DNMT activity kit (Cat # P-3009) and Epigenase 5mC-Hydroxylase TET Activity Kit (Cat # P-3086), respectively, as previously described by us and others [13,68].

### 4.4. Methylated DNA Immunoprecipitation (MeDIP)

In addition, we performed methylated DNA immunoprecipitation–PCR to determine overall DNA methylation status. We used a quick EpiQuik Tissue Methylated DNA immunoprecipitation kit (Cat # P-2020, EpiGentek, Farmingdale, NY, USA) followed by PCR according to the manufacturer’s protocol and as previously described [69].

### 4.5. Whole Genome Bisulfite Sequencing

Genomic DNA was isolated from 5 mg of tissue using a MasterPure™ DNA Purification Kit according to the manufacturer’s instructions. This kit employs non-enzymatically induced cell lysis followed by protein precipitation and subsequent nucleic acid isolation, which results in high yields of purified, high-molecular-weight DNA. The extracted DNA was resuspended in TE buffer and quantitated by fluorometry.

Bisulfite treatment of genomic DNA was performed using a Zymo EZ DNA Methylation Lightning Kit (Irvine, CA, USA). With this method, non-methylated cytosine nucleotides are converted to uracil and read as thymine (T) when sequenced. Methylated cytosines protected from conversion are still read as cytosine (C). Briefly, 50–100 ng of purified genomic DNA was treated with Zymo Lightning Conversion Reagent in a thermal cycler first for 8 min at 98 °C and then for 60 min at 54 °C. The bisulfite-treated DNA was purified on a spin column and used to prepare a sequencing library with an EpiGnome^TM^ Kit. In this procedure, bisulfite-treated single-stranded DNA was random-primed using a polymerase able to read uracil nucleotides to synthesize DNA strands containing a specific sequence tag at their 5′ ends. The 3′ ends of the newly synthesized DNA strands were then selectively tagged with a second specific sequence, resulting in DNA molecules di-tagged with known sequences at their 5′ and 3′ ends. Then using the PCR of the original DNA strand, these tags were used to add Illumina P7 and P5 adapters at the 5′ and 3′ ends, respectively. Because only the complement to the original bisulfite-treated DNA was used as the sequencing template, the resulting Read 1 would always be the same sequence as the original bisulfite-treated strands.

The EpiGnome libraries were diluted and loaded onto a cBot DNA Cluster Generation System. Once cluster generation was complete (after ~5 h), the flow cell was transferred to a HiSeq 2500 System (Illumina, Inc., San Diego, CA, USA) for sequencing using 75-bp paired-end reads. The HiSeq 2500 generates approximately 500 Gb of sequence data per flow cell or about 62 Gb per lane. Therefore, a single rat genome library can be run across two lanes of an eight-lane flow cell to generate approximately 120 Gb of data per sample. Additional sequencing can be completed for higher coverage.

Methylation analysis (www.epibio.com/docs/default-source/protocols/epignome-bioinformatics-user-guide.pdf?sfvrsn=2; accessed on 25 May 2021) was performed using Bismark (v2.4.3) [70]. FASTQ files were quality-filtered and adapter sequences trimmed using Trimmomatic [71]. A bisulfite-converted UCSC HG19 reference genome file was generated using Bowtie 2 (v2.2.5) [72], after which the EpiGnome library sequence data were aligned to the reference genome. Methylation information was extracted from the output *.sam file, and genome tracks were output for visualization (performed using www.broadinstitute.org/igv/home; accessed on 25 May 2021) and the reporting of downstream differential methylation calculations (performed using www.broadinstitute.org/igv/home; accessed on 25 May 2021) as previously described [10,12]. CpG sites with a coverage of 30 reads across all samples were selected for differential methylation analysis. Differential methylation loci (DML) analysis was performed with MethylKit (v2.0.1)—R package. If there were no biological duplicates in a group, then the Fisher exact test was used for DML calculation. Otherwise, a logistic regression was used. The genome was tiled with a window size of 1000 bp and the methylation information on those tiles was used for differential methylation region (DMR) analysis. Next, the methylation information of promoter regions and CpG was summarized, and the DM analysis was performed. Differential methylation results were filtered with a *p*-value < 0.05 and differential methylation level ≥ 25%. The filtered results were annotated with TSS information from Refseq. WGBS was performed by Genewiz, South Plainfield, NJ, USA.

### 4.6. RNA-Seq

RNA was isolated from aortas using Qiagen^®^ miRNeasy RNA isolation kits (Hilden, Germany). In brief, 20 mg samples from each tissue specimen were flash-frozen in liquid nitrogen and homogenized using a handheld homogenization pestle. Samples were then suspended in QIAzol Lysis reagent and further homogenized using a microtube homogenization mixer on ice. The subsequent steps were performed per the manufacturer’s protocol. RNA samples were eluted in 50 µL of RNase-free water. Adequate RNA yield was ensured using a DeNovix spectrophotometer, Wilmington, DE, USA. All samples yielded greater than 350 ng/µL of RNA, after which samples were stored at −80 °C until further use. RNA quality was assessed using a model 4200 TapeStation (Agilent Technologies, Santa Clara, CA, USA), and RNA quantity was determined using a Qubit 2 fluorometer (Life Technologies, Carlsbad, CA, USA). For each sample, 300 ng of RNA was used to construct a cDNA sequencing library with a TruSeq Stranded Total RNA Library Preparation Kit (Illumina, San Diego, CA, USA). The sequencing of paired-end reads (75 bp × 2) was performed in an Illumina NextSeq 550 system. Single-end sequencing was done at a depth of 30 million reads per replicate (*n* = 6). Sequence reads were trimmed to remove possible adapter sequences and nucleotides with poor quality using Trimmomatic v.0.36. The trimmed reads were mapped to the Rattus norvegicus Rnor6.0 reference genome available on ENSEMBL using the STAR aligner v.2.5.2b. The STAR aligner is a splice aligner that detects splice junctions and incorporates them to help align the entire read sequences. Unique gene hit counts were calculated by using featureCounts from the Subread package v.1.5.2. Only unique reads that fell within exon regions were counted. The hit counts are summarized using the gene_id feature. Low abundance genes with cpm < 10 were filtered and the gene count was normalized by the median of ratios method. After the extraction of gene hit counts, the gene hit counts table was used for downstream differential expression analysis. Differential expression of genes and transcripts or a comparison of gene expression was achieved in paired groups using the DESeq2 package on the RStudio platform (v1.3.1073, with R v4.1.0) as described previously [10,12,39]. The Wald test was used to generate *p*-values and log2 fold changes. The Benjamini–Hochberg method was used to generate an adjusted *p*-value. Genes with an adjusted *p*-value < 0.05 and absolute log2 fold change > 1 were called differentially expressed genes for each comparison. Significantly differentially expressed genes were clustered by their gene ontology and the enrichment of gene ontology terms was tested using Fisher’s exact test (GeneSCF v1.1-p2). RNAseq was performed by Genewiz, South Plainfield, NJ, USA.

### 4.7. Quantitative Real-Time PCR

Real-time PCR (RT-PCR) was used to analyze mRNA expression. Briefly, total RNA was extracted from aortas using a Qiagen miRNEasy kit (Cat # 217004). The quality and concentration of the input RNA were measured with a Synergy HT Take3 Microplate Reader (BioTek, Winooski, VT, USA), and cDNA was prepared using SuperScript IV. VILO Master Mix (Cat # 11756500, Invitrogen, Waltham, MA, USA) for mRNA. Quantitative PCR (qPCR) was performed in duplicate using TaqManTM Fast Advanced Master Mix (Cat # 44-445-57) for mRNA in a Mx3000p Real-Time PCR System (Stratagene, Santa Clara, CA, USA). The primers for the qPCR were purchased from Thermo Fisher Scientific/TaqMan. mRNA levels were normalized to internal control *Tuba1a*, and relative mRNA expression was reported.

### 4.8. UHPLC-MS Metabolomics

Snap-frozen tissue was ground to a powder (GenoGrinder, SPEX, Metuchen, NJ, USA) and extracted in ice-cold methanol:acetonitrile:water (5:3:2 *v*/*v*) at a concentration of 10 mg of tissue/1 mL of solution by vortexing for 30 min at 4 °C, followed by centrifugation at 15,000× *g* for 10 min at 4 °C. Twenty microliters of supernatant were collected from each extract for metabolomics analyses. Analyses were performed using a Vanquish UHPLC system coupled online to a Q Exactive mass spectrometer (Thermo Fisher, Bremen, Germany). Samples were resolved over a Kinetex C18 column (2.1 × 150 mm^2^, 1.7 µm; Phenomenex, Torrance, CA, USA) at 25 °C using a 3-min isocratic condition of 5% acetonitrile/95% water and 0.1% formic acid flowing at 250 µL/min, or using a 9-min gradient at 400 µL/min from 5 to 95% B (A: water; B: acetonitrile both phases coupled with 0.1% formic acid or 10 mM ammonium acetate for positive and negative ion mode, respectively). MS analysis and data elaboration were performed as described previously [73]. Metabolite assignment was performed using MAVEN v.3 (Princeton, NJ, USA) as described previously [74].

### 4.9. Measurement of G6PD Activity

G6PD activity was measured spectrophotometrically using an assay purchased from Cayman Chemicals, Ann Arbor, MI, USA. The standard assay buffer contained 1 mM MgCl_2_, 50 mM Tris, pH 8.10 (carefully adjusted with concentrated HCl), 0.1 mM NADP^+^, and 0.2 mM glucose-6-phosphate. Five-microliter aliquots (containing 5 μg of protein) of nuclear extract prepared from the aorta were pipetted into the wells of a 96-well plate (Fisher Scientific, Waltham, MA, USA) followed by the addition of 200 μL of the standard assay buffer. The absorbance at 339 nm was then measured using a plate reader (Synergy HT, Biotek) immediately and for up to 25 min at 50 s intervals. Background absorbance was corrected by subtracting the value of a blank containing no homogenate from all sample readings, and G6PD activity was determined quantitatively using a molar extinction coefficient of 6220 M^−1^cm^−1^.

### 4.10. Statistical Analysis

N represents the number of rats per group or biological replicates. All experiments (assays) were done in duplicate (technical replicates) to ensure the reliability of single values. Statistical analyses were performed using GraphPad Prism 9 software. Data are presented as a box and whiskers plot. Normality and outlier identification tests were performed, and outliers were removed. Statistical comparisons of samples were made for two groups with the Mann–Whitney test. To make comparisons among more than two groups, two-way ANOVA followed by Fisher’s LSD post-hoc test for multiple comparisons was used. The significance of RNAseq results was determined using the Benjamini–Hochberg method for multiple test correction (FDR). Values of *p* < 0.05 were considered significant.

## Data Availability

Data is contained within the article and Supplementary Materials. However, the data presented in this study are available on request from the corresponding author.

## Supplementary Materials

The following supporting information can be downloaded at: https://www.mdpi.com/article/10.3390/ijms242316727/s1.
