# Supplementary material for: G6PD Orchestrates Genome-Wide DNA Methylation and Gene Expression in the Vascular Wall
Source: Int J Mol Sci. 2023 Nov 24;24(23):16727. doi: 10.3390/ijms242316727 (PMC10706803; doi:10.3390/ijms242316727)
Supplement: Supplementary file 1 [file ijms-24-16727-s001.zip › ijms-2673378-supplementary.pdf]

Supplement

G6PD orchestrates genome-wide DNA methylation and gene expression in vascular wall

Christina Signoretti and Sachin A. Gupte

Department of Pharmacology, New York Medical College, Valhalla, NY, USA, 10595

Table S1. Differential Methylation Loci (DML) Analysis.

start	end	width	pvalue	qvalue	meth.diff	dist.to.feature	feature.name	feature.strand
1,944,206	1,944,206	1	0.00	0.05	-39.38	5,788	ENSRNOT00000065646	+
2,295,592	2,295,592	1	0.00	0.02	25.58	-221,697	ENSRNOT00000061999	-
2,398,382	2,398,382	1	0.00	0.04	26.28	229,366	ENSRNOT00000022003	-
2,557,383	2,557,383	1	0.00	0.03	33.93	70,365	ENSRNOT00000022003	-
2,613,222	2,613,222	1	0.00	0.03	-32.76	14,526	ENSRNOT00000022003	-
2,730,124	2,730,124	1	0.00	0.00	-34.48	9,826	ENSRNOT00000080492	+
2,885,946	2,885,946	1	0.00	0.00	50.19	408	ENSRNOT00000079768	-
3,240,075	3,240,075	1	0.00	0.04	-30.04	-353,723	ENSRNOT00000079768	-
3,589,847	3,589,847	1	0.00	0.04	-28.75	173,546	ENSRNOT00000034744	-
3,861,394	3,861,394	1	0.00	0.03	-38.45	-12,315	ENSRNOT00000045301	-
3,861,410	3,861,410	1	0.00	0.00	-55.30	-12,331	ENSRNOT00000045301	-
3,960,609	3,960,609	1	0.00	0.04	-27.27	-7,928	ENSRNOT00000086489	-
3,964,857	3,964,857	1	0.00	0.04	-25.53	-12,176	ENSRNOT00000086489	-
4,017,921	4,017,921	1	0.00	0.01	-26.83	-6,411	ENSRNOT00000040559	-
4,020,028	4,020,028	1	0.00	0.02	-34.43	-8,518	ENSRNOT00000040559	-
4,126,681	4,126,681	1	0.00	0.01	33.71	-115,171	ENSRNOT00000040559	-
4,151,858	4,151,858	1	0.00	0.03	34.87	-140,348	ENSRNOT00000040559	-
4,344,179	4,344,179	1	0.00	0.03	30.83	12,314	ENSRNOT00000087887	-
4,476,858	4,476,858	1	0.00	0.02	-29.41	73,911	ENSRNOT00000061954	-
4,650,509	4,650,509	1	0.00	0.04	-32.85	2,702	ENSRNOT00000019121	-
4,866,269	4,866,269	1	0.00	0.01	-31.37	134,333	ENSRNOT00000079051	+
5,718,135	5,718,135	1	0.00	0.04	42.57	-22,010	ENSRNOT00000091247	+
5,855,555	5,855,555	1	0.00	0.01	30.23	115,412	ENSRNOT00000091247	+
6,427,578	6,427,578	1	0.00	0.04	-44.44	-12,478	ENSRNOT00000088346	+
6,597,851	6,597,851	1	0.00	0.04	-37.89	157,797	ENSRNOT00000088346	+
6,857,718	6,857,718	1	0.00	0.04	-36.10	45,769	ENSRNOT00000086574	-
6,922,099	6,922,099	1	0.00	0.02	-41.33	-18,614	ENSRNOT00000086574	-
6,922,389	6,922,389	1	0.00	0.00	-44.44	-18,904	ENSRNOT00000086574	-
6,922,461	6,922,461	1	0.00	0.00	-41.17	-18,976	ENSRNOT00000086574	-
6,922,562	6,922,562	1	0.00	0.04	-34.98	-19,077	ENSRNOT00000086574	-
6,953,696	6,953,696	1	0.00	0.04	39.14	16,345	ENSRNOT00000016273	-
7,045,205	7,045,205	1	0.00	0.02	42.15	19,666	ENSRNOT00000019983	-
7,266,162	7,266,162	1	0.00	0.04	-38.39	13,814	ENSRNOT00000030329	+
7,923,873	7,923,873	1	0.00	0.04	38.16	114,793	ENSRNOT00000046539	-
7,998,642	7,998,642	1	0.00	0.03	-40.01	40,024	ENSRNOT00000046539	-
8,025,503	8,025,503	1	0.00	0.01	-41.84	13,163	ENSRNOT00000046539	-
8,028,447	8,028,447	1	0.00	0.02	-40.28	10,219	ENSRNOT00000046539	-
8,139,603	8,139,603	1	0.00	0.02	-37.07	-100,939	ENSRNOT00000046539	-
8,139,628	8,139,628	1	0.00	0.00	-39.48	-100,964	ENSRNOT00000046539	-
8,355,461	8,355,461	1	0.00	0.02	-38.33	44,885	ENSRNOT00000015131	+
8,634,480	8,634,480	1	0.00	0.01	33.29	116,719	ENSRNOT00000030511	-

9,379,556	9,379,556	1	0.00	0.04	32.43	177,673	ENSRNOT00000089566	+
9,616,061	9,616,061	1	0.00	0.04	34.23	-232,243	ENSRNOT00000085963	+
10,911,329	10,911,329	1	0.00	0.01	-32.00	135,691	ENSRNOT00000078557	+
11,303,445	11,303,445	1	0.00	0.02	-33.45	-454,786	ENSRNOT00000072832	+
11,906,939	11,906,939	1	0.00	0.04	-26.54	-347	ENSRNOT00000089050	-
11,906,942	11,906,942	1	0.00	0.01	-31.48	-350	ENSRNOT00000089050	-
11,907,557	11,907,557	1	0.00	0.03	-38.99	-965	ENSRNOT00000089050	-
11,907,566	11,907,566	1	0.00	0.03	-38.53	-974	ENSRNOT00000089050	-
11,907,578	11,907,578	1	0.00	0.03	-37.84	-986	ENSRNOT00000089050	-
11,910,100	11,910,100	1	0.00	0.02	-29.81	-3,508	ENSRNOT00000089050	-
11,910,229	11,910,229	1	0.00	0.00	-34.06	-3,637	ENSRNOT00000089050	-
11,910,235	11,910,235	1	0.00	0.01	-32.26	-3,643	ENSRNOT00000089050	-
11,910,239	11,910,239	1	0.00	0.00	-34.37	-3,647	ENSRNOT00000089050	-
11,910,297	11,910,297	1	0.00	0.01	-29.71	-3,705	ENSRNOT00000089050	-
11,910,302	11,910,302	1	0.00	0.00	-30.90	-3,710	ENSRNOT00000089050	-
11,913,161	11,913,161	1	0.00	0.01	-30.99	2,463	ENSRNOT00000081180	-
11,913,167	11,913,167	1	0.00	0.02	-31.92	2,457	ENSRNOT00000081180	-
11,915,830	11,915,830	1	0.00	0.03	-26.64	-208	ENSRNOT00000081180	-
11,930,613	11,930,613	1	0.00	0.00	31.73	-14,991	ENSRNOT00000081180	-
11,966,723	11,966,723	1	0.00	0.05	-25.14	2,888	ENSRNOT00000074325	+
12,057,590	12,057,590	1	0.00	0.02	44.64	50,931	ENSRNOT00000093439	+
12,664,205	12,664,205	1	0.00	0.03	26.53	93,755	ENSRNOT00000085926	-
12,732,798	12,732,798	1	0.00	0.03	-45.76	25,162	ENSRNOT00000085926	-
12,868,567	12,868,567	1	0.00	0.01	-39.66	-38,333	ENSRNOT00000082011	+
12,868,573	12,868,573	1	0.00	0.03	-36.64	-38,327	ENSRNOT00000082011	+
13,187,371	13,187,371	1	0.00	0.01	-43.05	-11,496	ENSRNOT00000084870	-
14,357,010	14,357,010	1	0.00	0.03	41.35	55,798	ENSRNOT00000074583	-
14,442,347	14,442,347	1	0.00	0.03	43.86	-29,541	ENSRNOT00000074583	-
14,469,164	14,469,164	1	0.00	0.04	-39.27	-38,793	ENSRNOT00000088269	+
14,601,386	14,601,386	1	0.00	0.03	-44.79	73,671	ENSRNOT00000077965	+
14,629,570	14,629,570	1	0.00	0.02	39.35	101,855	ENSRNOT00000077965	+
15,063,759	15,063,759	1	0.00	0.04	-38.89	1,328	ENSRNOT00000016286	+
15,438,580	15,438,580	1	0.00	0.03	-33.55	25,978	ENSRNOT00000067070	+
15,544,558	15,544,558	1	0.00	0.00	-43.77	-3,820	ENSRNOT00000091752	-
15,567,054	15,567,054	1	0.00	0.01	-39.66	-26,316	ENSRNOT00000091752	-
15,588,636	15,588,636	1	0.00	0.04	-41.46	-32,018	ENSRNOT00000017401	+
15,588,680	15,588,680	1	0.00	0.03	-38.72	-31,974	ENSRNOT00000017401	+
15,774,219	15,774,219	1	0.00	0.02	-41.99	-3,955	ENSRNOT00000079244	+
15,827,296	15,827,296	1	0.00	0.05	-33.83	-7,484	ENSRNOT00000079069	+
15,842,254	15,842,254	1	0.00	0.02	-41.83	7,377	ENSRNOT00000083012	+
15,852,320	15,852,320	1	0.00	0.00	-51.43	17,443	ENSRNOT00000083012	+
15,909,060	15,909,060	1	0.00	0.01	-40.14	74,183	ENSRNOT00000083012	+
15,978,266	15,978,266	1	0.00	0.02	-30.95	143,389	ENSRNOT00000083012	+
15,984,945	15,984,945	1	0.00	0.01	-44.91	150,068	ENSRNOT00000083012	+
15,985,572	15,985,572	1	0.00	0.02	-41.03	150,695	ENSRNOT00000083012	+

16,050,531	16,050,531	1	0.00	0.04	-35.36	153,308	ENSRNOT00000018556	-
16,109,889	16,109,889	1	0.00	0.01	38.66	93,950	ENSRNOT00000018556	-
16,201,888	16,201,888	1	0.00	0.00	-38.06	1,951	ENSRNOT00000018556	-
16,327,367	16,327,367	1	0.00	0.01	46.11	-62,460	ENSRNOT00000089467	+
16,687,965	16,687,965	1	0.00	0.03	-40.54	-149	ENSRNOT00000091376	-
16,823,431	16,823,431	1	0.00	0.05	-33.76	4,183	ENSRNOT00000086756	+
16,823,769	16,823,769	1	0.00	0.01	-41.11	4,521	ENSRNOT00000086756	+
17,931,907	17,931,907	1	0.00	0.00	-51.66	126,149	ENSRNOT00000020988	-
18,035,375	18,035,375	1	0.00	0.02	-33.37	22,681	ENSRNOT00000020988	-
18,056,118	18,056,118	1	0.00	0.03	-37.00	1,938	ENSRNOT00000020988	-
18,056,121	18,056,121	1	0.00	0.01	-40.51	1,935	ENSRNOT00000020988	-
18,239,217	18,239,217	1	0.00	0.03	39.68	-114,596	ENSRNOT00000029394	+
18,256,589	18,256,589	1	0.00	0.01	-27.03	-97,224	ENSRNOT00000029394	+
18,634,102	18,634,102	1	0.00	0.01	-44.68	56,067	ENSRNOT00000088635	+

Table S2. Differential Methylation Region (DMR) Analysis by Using Tiled Windows.

start	end	width	pvalue	qvalue	meth.diff	dist.to.feature	feature.name	feature.strand
187,001	188,000	1,000	0.00	0.00	35.69	-208,701	ENSRNOT00000044187	+
444,001	445,000	1,000	0.00	0.00	-25.77	47,162	ENSRNOT00000093216	+
3,260,001	3,261,000	1,000	0.00	0.00	-34.21	-373,649	ENSRNOT00000079768	-
4,476,001	4,477,000	1,000	0.00	0.00	-29.41	73,769	ENSRNOT00000061954	-
6,033,001	6,034,000	1,000	0.00	0.00	25.39	-4,602	ENSRNOT00000082128	+
6,122,001	6,123,000	1,000	0.00	0.00	25.63	83,401	ENSRNOT00000082128	+
6,177,001	6,178,000	1,000	0.00	0.02	-26.20	101,794	ENSRNOT00000091348	-
7,411,001	7,412,000	1,000	0.00	0.00	53.73	31,864	ENSRNOT00000088558	-
7,509,001	7,510,000	1,000	0.00	0.00	27.03	-28,177	ENSRNOT00000048754	-
7,543,001	7,544,000	1,000	0.00	0.00	-37.43	-62,177	ENSRNOT00000048754	-
8,947,001	8,948,000	1,000	0.00	0.00	-27.13	13,101	ENSRNOT00000016215	+
9,798,001	9,799,000	1,000	0.00	0.01	30.77	-49,304	ENSRNOT00000085963	+
11,959,001	11,960,000	1,000	0.00	0.00	-26.04	-2,751	ENSRNOT00000088595	+
15,588,001	15,589,000	1,000	0.00	0.00	-26.51	-31,654	ENSRNOT00000017401	+
16,966,001	16,967,000	1,000	0.00	0.00	-30.33	946	ENSRNOT00000084087	-
25,023,001	25,024,000	1,000	0.00	0.04	29.17	-40,396	ENSRNOT00000074143	-
25,073,001	25,074,000	1,000	0.00	0.00	40.70	-90,396	ENSRNOT00000074143	-
28,460,001	28,461,000	1,000	0.00	0.03	-29.46	4,951	ENSRNOT00000030327	+
29,426,001	29,427,000	1,000	0.00	0.00	-34.76	-5,153	ENSRNOT00000019436	+
29,565,001	29,566,000	1,000	0.00	0.00	25.69	132,828	ENSRNOT00000083276	+
30,647,001	30,648,000	1,000	0.00	0.00	-42.60	18,754	ENSRNOT00000083664	-
30,723,001	30,724,000	1,000	0.00	0.00	-32.49	41,321	ENSRNOT00000015395	+
34,950,001	34,951,000	1,000	0.00	0.00	39.05	76,372	ENSRNOT00000087018	-
37,601,001	37,602,000	1,000	0.00	0.00	-40.41	-79,260	ENSRNOT00000084864	+
37,725,001	37,726,000	1,000	0.00	0.00	-34.62	152	ENSRNOT00000071842	-
37,884,001	37,885,000	1,000	0.00	0.02	28.47	1,676	ENSRNOT00000024149	-
38,955,001	38,956,000	1,000	0.00	0.00	-25.66	-72,506	ENSRNOT00000086932	-

40,190,001	40,191,000	1,000	0.00	0.00	-26.12	64,708	ENSRNOT00000081556	-
40,213,001	40,214,000	1,000	0.00	0.00	26.67	41,708	ENSRNOT00000081556	-
41,564,001	41,565,000	1,000	0.00	0.00	-27.88	-25,495	ENSRNOT00000089984	+
41,698,001	41,699,000	1,000	0.00	0.00	44.56	55,653	ENSRNOT00000047472	-
42,779,001	42,780,000	1,000	0.00	0.00	33.66	-37,451	ENSRNOT00000045014	-
43,252,001	43,253,000	1,000	0.00	0.00	35.70	-201,804	ENSRNOT00000086685	+
43,774,001	43,775,000	1,000	0.00	0.02	25.30	56,933	ENSRNOT00000082931	-
44,449,001	44,450,000	1,000	0.00	0.00	-30.28	2,237	ENSRNOT00000030132	+
45,829,001	45,830,000	1,000	0.00	0.00	-26.50	-459	ENSRNOT00000089275	-
47,927,001	47,928,000	1,000	0.00	0.00	31.56	-5,415	ENSRNOT00000025794	-
48,384,001	48,385,000	1,000	0.00	0.00	29.33	-23,741	ENSRNOT00000023116	-
48,534,001	48,535,000	1,000	0.00	0.00	-25.09	24,163	ENSRNOT00000080352	-
50,487,001	50,488,000	1,000	0.00	0.00	-28.60	-340,135	ENSRNOT00000089149	+
52,531,001	52,532,000	1,000	0.00	0.00	30.49	12,451	ENSRNOT00000043474	-
54,192,001	54,193,000	1,000	0.00	0.00	-25.49	-64,433	ENSRNOT00000087624	-
54,561,001	54,562,000	1,000	0.00	0.03	-26.54	-54,247	ENSRNOT00000082407	-
54,955,001	54,956,000	1,000	0.00	0.00	-26.00	-71,440	ENSRNOT00000092209	-
55,697,001	55,698,000	1,000	0.00	0.00	25.54	-9,399	ENSRNOT00000089548	+
55,764,001	55,765,000	1,000	0.00	0.00	32.23	56,604	ENSRNOT00000089548	+
55,857,001	55,858,000	1,000	0.00	0.00	29.20	37,637	ENSRNOT00000029537	-
55,925,001	55,926,000	1,000	0.00	0.00	-25.18	-29,366	ENSRNOT00000029537	-
58,140,001	58,141,000	1,000	0.00	0.02	-25.11	140,507	ENSRNOT00000084574	+
58,462,001	58,463,000	1,000	0.00	0.01	-28.75	-116,552	ENSRNOT00000090895	-
58,564,001	58,565,000	1,000	0.00	0.00	33.53	-218,552	ENSRNOT00000090895	-
58,865,001	58,866,000	1,000	0.00	0.00	-26.59	77,625	ENSRNOT00000079970	+
59,029,001	59,030,000	1,000	0.00	0.04	28.80	-99,952	ENSRNOT00000017165	+
60,910,001	60,911,000	1,000	0.00	0.00	-29.69	25,241	ENSRNOT00000088689	+
61,305,001	61,306,000	1,000	0.00	0.00	31.77	-8,071	ENSRNOT00000077642	+
61,645,001	61,646,000	1,000	0.00	0.00	26.64	-18,548	ENSRNOT00000038190	-
62,440,001	62,441,000	1,000	0.00	0.00	32.14	-77,449	ENSRNOT00000089634	-
62,447,001	62,448,000	1,000	0.00	0.00	44.64	-84,449	ENSRNOT00000089634	-
63,331,001	63,332,000	1,000	0.00	0.01	-26.04	-1,284	ENSRNOT00000086294	+
64,173,001	64,174,000	1,000	0.00	0.00	-28.69	1,100	ENSRNOT00000091288	-
64,821,001	64,822,000	1,000	0.00	0.00	-29.09	8,954	ENSRNOT00000087034	-
64,998,001	64,999,000	1,000	0.00	0.03	27.12	59,503	ENSRNOT00000092003	-
66,323,001	66,324,000	1,000	0.00	0.01	25.83	-1,360	ENSRNOT00000074234	-
66,592,001	66,593,000	1,000	0.00	0.00	37.04	46,206	ENSRNOT00000082190	-
67,774,001	67,775,000	1,000	0.00	0.00	29.46	31,053	ENSRNOT00000049350	+
70,239,001	70,240,000	1,000	0.00	0.03	25.64	-3,911	ENSRNOT00000075745	-
71,234,001	71,235,000	1,000	0.00	0.00	30.45	-10,337	ENSRNOT00000086416	-
71,825,001	71,826,000	1,000	0.00	0.01	25.21	-18,369	ENSRNOT00000044754	+
72,814,001	72,815,000	1,000	0.00	0.00	27.26	3,457	ENSRNOT00000092117	+
73,146,001	73,147,000	1,000	0.00	0.03	-25.86	-24,917	ENSRNOT00000073464	-
73,328,001	73,329,000	1,000	0.00	0.00	27.04	15,956	ENSRNOT00000039823	-
74,008,001	74,009,000	1,000	0.00	0.04	25.15	-46,570	ENSRNOT00000035435	-

74,247,001	74,248,000	1,000	0.00	0.00	25.51	-3,168	ENSRNOT00000040441	+
77,441,001	77,442,000	1,000	0.00	0.00	-27.21	93,682	ENSRNOT00000018559	-
78,224,001	78,225,000	1,000	0.00	0.00	25.77	0	ENSRNOT00000075700	-
79,030,001	79,031,000	1,000	0.00	0.00	41.50	0	ENSRNOT00000088925	+
79,152,001	79,153,000	1,000	0.00	0.01	-26.47	-28,856	ENSRNOT00000037132	+
80,450,001	80,451,000	1,000	0.00	0.00	-26.29	20,528	ENSRNOT00000023867	-
81,195,001	81,196,000	1,000	0.00	0.00	26.36	-1,273	ENSRNOT00000026268	-
82,266,001	82,267,000	1,000	0.00	0.00	-25.74	0	ENSRNOT00000027910	-
82,344,001	82,345,000	1,000	0.00	0.00	-25.48	0	ENSRNOT00000090629	-
82,385,001	82,386,000	1,000	0.00	0.00	-26.54	13,613	ENSRNOT00000088115	+
83,316,001	83,317,000	1,000	0.00	0.01	-26.47	26,259	ENSRNOT00000040989	+
84,415,001	84,416,000	1,000	0.00	0.00	-26.00	3,276	ENSRNOT00000025303	+
84,421,001	84,422,000	1,000	0.00	0.00	-27.19	9,276	ENSRNOT00000025303	+
84,422,001	84,423,000	1,000	0.00	0.00	-26.71	10,276	ENSRNOT00000025303	+
86,699,001	86,700,000	1,000	0.00	0.00	29.60	45,778	ENSRNOT00000050523	+
86,799,001	86,800,000	1,000	0.00	0.00	28.95	3,615	ENSRNOT00000080451	-
87,138,001	87,139,000	1,000	0.00	0.00	25.67	8,309	ENSRNOT00000027773	-
87,637,001	87,638,000	1,000	0.00	0.00	26.18	-12,208	ENSRNOT00000077161	+
89,540,001	89,541,000	1,000	0.00	0.00	33.84	-792	ENSRNOT00000028644	-
89,651,001	89,652,000	1,000	0.00	0.00	-55.28	1,366	ENSRNOT00000085953	+
90,134,001	90,135,000	1,000	0.00	0.03	31.43	-2,626	ENSRNOT00000076885	-
90,455,001	90,456,000	1,000	0.00	0.01	25.42	64,013	ENSRNOT00000028698	-
90,567,001	90,568,000	1,000	0.00	0.00	-27.70	-46,658	ENSRNOT00000078598	-
91,276,001	91,277,000	1,000	0.00	0.00	-28.27	19,657	ENSRNOT00000028703	-
91,366,001	91,367,000	1,000	0.00	0.00	-30.87	2,510	ENSRNOT00000014517	+
91,386,001	91,387,000	1,000	0.00	0.00	-26.01	22,510	ENSRNOT00000014517	+
91,391,001	91,392,000	1,000	0.00	0.00	-26.24	27,510	ENSRNOT00000014517	+
91,433,001	91,434,000	1,000	0.00	0.00	-25.80	0	ENSRNOT00000015205	+

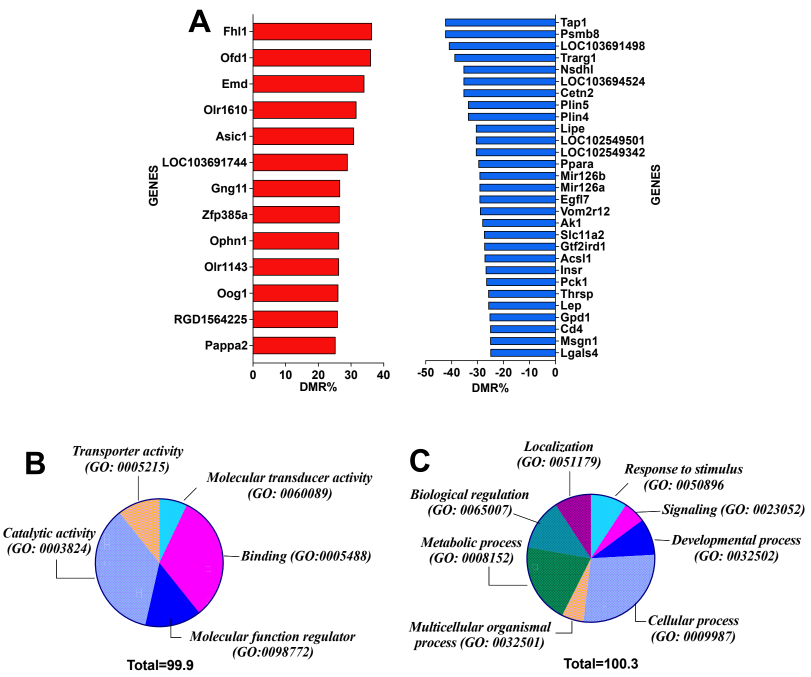

Figure S1. (A) A few significantly hyper- (red bar) and hypo (blue bar)-methylated (>25%) promoter regions are shown. Pie charts show GO term enrichment of (B) hypermethylated and (C) hypomethylated promoter regions identified in WT *versus* G6PD^{S188F} rats.
